# A convolutional neural network-based screening tool for X-ray serial crystallography

**DOI:** 10.1107/S1600577518004873

**Published:** 2018-04-24

**Authors:** Tsung-Wei Ke, Aaron S. Brewster, Stella X. Yu, Daniela Ushizima, Chao Yang, Nicholas K. Sauter

**Affiliations:** a International Computer Science Institute, University of California Berkeley, Berkeley, CA 94704, USA; b Molecular Biophysics and Integrated Bioimaging Division, Lawrence Berkeley National Laboratory, Berkeley, CA 94720, USA; c Computational Research Division, Lawrence Berkeley National Laboratory, Berkeley, CA 94720, USA; d Berkeley Institute for Data Science, University of California Berkeley, Berkeley, CA 94704, USA

**Keywords:** convolutional neural networks, deep learning, serial crystallography, X-ray free-electron laser, macromolecular structure

## Abstract

A new tool is introduced for screening macromolecular X-ray crystallography diffraction images produced at an X-ray free-electron laser light source. Based on a data-driven deep learning approach, the proposed tool executes a convolutional neural network to detect Bragg spots. Automatic image processing algorithms described can enable the classification of large data sets, acquired under realistic conditions consisting of noisy data with experimental artifacts. Outcomes are compared for different data regimes, including samples from multiple instruments and differing amounts of training data for neural network optimization.

## Abstract 2

Deep learning provides one possible avenue to reduce the data stream generated by serial macromolecular X-ray crystallography. Convolutional neural networks can be trained to recognize the presence or absence of Bragg spots, forming a criterion to veto events prior to downstream data processing.

## 1. Introduction   

**Figure 1:**
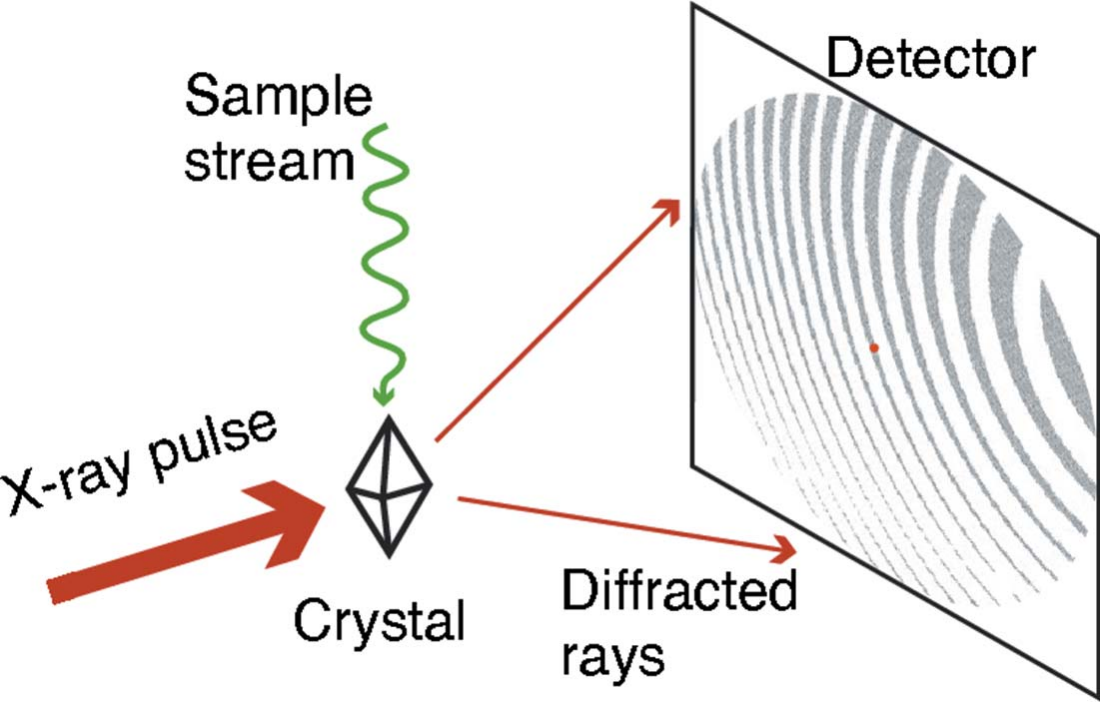
Generic scheme depicting the detection of Bragg spots in an XFEL crystallography experiment. After the diffraction is recorded (within several tens of femtoseconds), the X-ray pulse destroys the crystal sample, which is therefore replenished after each shot by any one of numerous sample delivery mechanisms.

The recent introduction of X-ray free-electron laser (XFEL) light sources has made it possible to determine three-dimensional macromolecular structures from crystal diffraction patterns, acquired before radiation damage processes occur, that are generated from samples equilibrated at room temperature. This ‘diffract-before-destroy’ approach, enabled by femtosecond X-ray pulses, has allowed the examination of proteins in states that closely resemble the biologically relevant form, where metal atom co-factors remain in their native valence state (Alonso-Mori *et al.*, 2012 ▸), and amino acid residues populate the full ensemble of rotational states important for the function of a given protein (Keedy *et al.*, 2015 ▸). The necessity of replacing the crystal after every shot (serial crystallography) has led to the development of entirely new methods for sample delivery (Weierstall *et al.*, 2012 ▸, 2014 ▸; Sierra *et al.*, 2012 ▸, 2016 ▸; Sugahara *et al.*, 2015 ▸; Fuller *et al.*, 2017 ▸; Roedig *et al.*, 2017 ▸; Orville, 2017 ▸), since the assembly of a full dataset requires the collection of 10^2^–10^5^ diffraction patterns (Fig. 1 ▸).

In serial crystallography, as with traditional single-crystal diffraction protocols, it is important to arrange for data processing capabilities that produce real time feedback, in order to understand the characteristics of the experimental results. Full data analysis, on a time scale significantly shorter than the data collection, permits key indicators to be monitored so that experimental parameters can be adjusted before the available sample and allotted beam time are exhausted. Such indicators include the rate of increase in reciprocal space coverage (*i.e.* how fast the multiplicity and completeness increase over time), the limiting resolution of the diffraction pattern, the crystalline disorder or mosaicity, and the extent to which the reaction has been triggered, for those experiments where the macromolecule is being followed as a function of time. Providing for sufficient computing resources is a continuing challenge (Thayer *et al.*, 2016 ▸), with current-year experiments at the Linac Coherent Light Source (LCLS) able to draw upon either a dedicated 288-core Linux cluster^1^, or a shared supercomputer facility such as the National Energy Research Scientific Computing Center (NERSC)^2^. As data collection capacity continues to increase, it is important to assure that the data production rate does not exceed the network bandwidth for transmission to such facilities. To this end, it is critical to develop the ability to veto certain diffraction events (such as those images that contain no Bragg spots) at the time and place of data collection, so that network and data processing resources are not overloaded by data with little value. Ideally, a screening tool that can quickly distinguish a ‘hit’ (an image with Bragg spots) from a ‘miss’ plays a major role in data reduction, so that only the hits are propagated to data analysis and storage.

In this paper, we propose such a screening tool using a data-driven deep learning approach on a convolutional neural network (CNN) model. Compared with traditional image processing techniques, the CNN has the advantage of being able to encode both visual perception and human knowledge that are difficult to quantify precisely. A knowledgeable X-ray crystallographer can readily recognize a pattern of Bragg spots, if one exists. Even when the intensity of the spots is low relative to the background, the expert will draw upon other heuristics such as the Bragg spot areas and shapes, the reciprocal lattice pattern, the density of spots, and the distribution of the brightest spots at low scattering angles, all of which may be difficult to express in a precise mathematical fashion. The use of a CNN can exploit these heuristics through supervised learning, the process that optimizes the CNN’s parameters and thus encodes a highly nonlinear mathematical model that represents the expert knowledge.

Other types of neural networks have been used to screen X-ray diffraction images in the past (Becker & Streit, 2014 ▸; Berntson *et al.*, 2003 ▸). In Becker & Streit (2014 ▸), a number of image attributes, such as the maximum pixel intensity, mean pixel intensity and the standard deviation, are calculated for each image and used as the inputs to a single-layer feed-forward neural network to classify an image as good or bad. However, since these attributes do not contain any information about the presence of Bragg spots, this type of neural network may only allow us to remove a subset of the images that have poor contrast or that contain a significant amount of noise or artifacts. A number of sophisticated preprocessing algorithms are used by Berntson *et al.* (2003 ▸) to analyze the distribution and patterns of high intensity pixels, for input into a feed-forward neural network. In contrast, the optimization of our CNN requires us only to provide labeled data, *i.e.* a set of previously analyzed images that are classified by an expert.

A potential deliverable is that a CNN, properly trained on a small number of datasets, can be used to screen new measurements in real time. If implemented on dedicated hardware such as an energy-efficient neuromorphic chip (Merolla *et al.*, 2014 ▸; Esser *et al.*, 2016 ▸), this type of screening procedure could in the future be coupled directly with the imaging detector as part of the data acquisition system.

## 2. Methodology   

### 2.1. Convolutional neural network   

Deep learning has become a powerful tool for machine learning that can be used to leverage existing human knowledge present in properly annotated data, to perform a wide variety of cognitive and inference tasks (Goodfellow *et al.*, 2016 ▸; Geron, 2016 ▸; LeCun *et al.*, 2015 ▸). CNNs are widely used to solve numerous computer vision problems, *e.g.* image classification and object tracking. These artificial neural networks are composed of several layers of neurons, mediated by connections between successive input and output layers. In the connection layer, each output neuron at one level is connected to some or all of the input neurons at the next level. Also, different spatial locations in one layer may share the same connection patterns. Each connection unit acts as a linear or a non-linear operator defined in terms of a set of parameters. These parameters are optimized jointly in an iterative learning process that minimizes the discrepancy between the nonlinear output of the network and the desired output labels.

**Figure 2:**
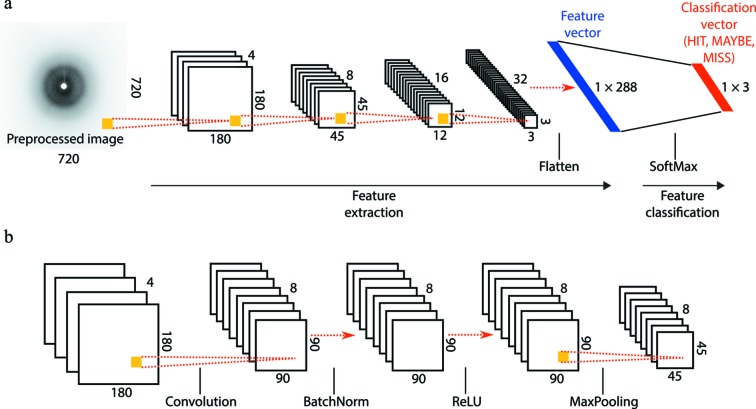
The proposed CNN for screening X-ray images consists of four sets of convolution / batch normalization / rectification (ReLU) / downsampling (MaxPooling) layers, as detailed in §2.1. (*a*) Numbers and the sizes of the output feature response maps produced by each of the filter layers. The resulting feature map is concatenated (flattened) into a one-dimensional feature vector, and fed into the feature classification stage. (*b*) Details of the particular sequence of filters that is responsible for the transformation from the 4 × 180 × 180 image stack to the 8 × 45 × 45 stack.

For the present problem of classifying X-ray crystallography diffraction images, we adopt a network with a structure similar to that of AlexNet (Krizhevsky *et al.*, 2012 ▸), the first successful deep learning network used for object recognition over large-scale real-world datasets (Deng *et al.*, 2009 ▸). A similar implementation was also used recently to calibrate the rotation axis in X-ray computed tomography data (Yang *et al.*, 2017 ▸). The CNN employs two steps (Fig. 2 ▸): feature extraction and feature classification. Feature extraction takes a gray-scale image stored as a two-dimensional array of size 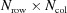 as its input, and maps the two-dimensional image to a feature vector of size 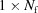 through multiple neuron layers, where 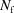 is the number of high-level features described by the output (here, 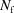 = 288). In the second step, the feature vector is mapped to a probabilistic classification vector of size 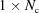, where 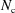 is the number of classification categories. This paper considers three categories: ‘Hit’, ‘Maybe’ or ‘Miss’, with ‘Maybe’ indicating that a given image contains only a relatively small number of Bragg spots. An output vector [0.6, 0.3, 0.1] indicates that the CNN predicts the input image to have a 60% chance of being a Hit, 30% chance of being Maybe and 10% chance of being a Miss. To assign the input image to a single class, we add the Hit or Maybe probabilities and then use a winner-takes-all approach that turns the soft classification vector (*e.g.* 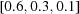) into a hard yes/no decision, which in this particular case is 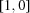: a ‘Hit’.

#### 2.1.1. Feature extraction   

The purpose of feature extraction is to produce a final 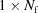 vector (Fig. 2*a* ▸) that succinctly summarizes the relevant information in the original image. This vector is produced from the input image by the application of four successive computational filters, each reducing the spatial resolution. Each filter consists of convolution, batch normalization, rectification and downsampling operations (Fig. 2*b* ▸) that are described as follows.

(i) A convolutional layer that takes 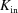 images as the input and produces 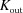 images as the output contains 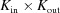 two-dimensional convolution kernels 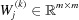, 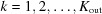, 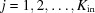, that are local and translation invariant. By convolving the input image 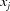 with kernels 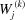, we obtain 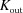 *neural activations* at that layer,
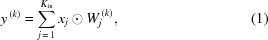
where 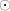 is used to denote the convolution operation. The size of the output image 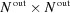 depends on the size of the input image 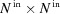, the kernel size 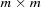, as well as the stride *s* used during the convolution, which is defined to be the number of pixels the kernel moves in each direction as the moving average is computed in the convolution process. With zero-padding at the image boundaries, 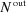 ≃ 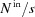, where 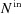 is the square edge size of the input image. Earlier convolutional layers tend to extract and separate local features such as edges and corners from *x* into 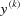. These features are then used as input to the next layer.

In our implementation, we set the convolutional kernel size to 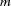 = 3 and stride to 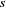 = 2. If we consider 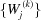, 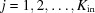, as a single three-dimensional convolutional kernel with 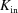 two-dimensional slices, then the number of convolutional kernels used in each of the four iterations is set to 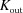 = 4, 8, 16 and 32, respectively.

To illustrate this computational workflow in Fig. 2(*b*) ▸, the first filter (labeled ‘Convolution’) depicts the action of 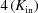 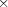 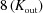 = 32 total two-dimensional convolution kernels each of size 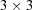, thus mapping four input images to eight output images. The size reduction from 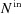 = 180 to 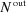 = 90 is a consequence of the stride size (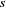 = 2) with which the kernel is moved along the input image during the convolution process. The yellow square depicts the first kernel, 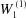, which results in the contribution of a 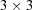 square of pixels from the first input image to a single pixel in the first output image.

(ii) Batch normalization [BN (Ioffe & Szegedy, 2015 ▸)] is a layer that rescales each image based on the distribution of image intensities within a subset of training images often referred to as a mini-batch. A mini-batch is a random miniature version of the entire training set, and the CNN is optimized over the exposure to one mini-batch at a time. The rescaling parameters are determined as part of the training process. It has been observed that rescaling allows the training process to converge faster.

(iii) The rectified linear unit layer [ReLU (Nair & Hinton, 2010 ▸)] applies an activation function to an input neuron at each pixel location. The activation function is defined as 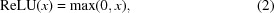
where *x* is the intensity of a pixel. It controls how much information is passed through the network.

(iv) A pooling layer performs a down-sampling process that coalesces an 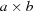 patch of pixels into a single pixel with an intensity defined as either the average (average pooling) or the maximum (max pooling) intensity among all pixels within the patch. If we assume the input *x* is a square image of size 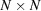, and 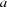 = 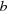, then the output *y* of the pooling operation is of size 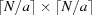. In our implementation, the downsampling rate is set to 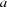 = 2, and to illustrate this in Fig. 2(*b*) ▸ the yellow square in the ‘MaxPooling’ filter represents the contribution of a 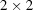 square of pixels from the input image to a single pixel in the output image.

The 32 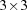 images that emerge from the fourth successive filter in Fig. 2(*a*) ▸ are flattened into a single one-dimensional vector before being sent into the classification layer.

#### 2.1.2. Pattern classification   

Once important features are extracted from the input image and held in a 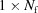 feature vector, the feature classifier maps them to a 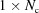 classification vector 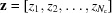, through one fully connected layer. Unlike the convolutional layer where the output neurons in one layer share the same local connection patterns with the input neurons, for a fully connected layer each output neuron has its own connection patterns to all the input neurons. Next, the real-valued 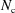 output vector is mapped into a probabilistic classification vector using the so-called SoftMax layer. The SoftMax layer maps the classification vector 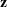 to a proper categorical distribution, by element-wise exponentiation and vector-wise normalization, 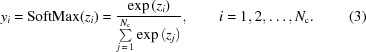
This operation turns every component of 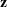, which may have a positive or negative value, into a nonnegative number between 0 and 1. The vector *y* is normalized to have a unit sum. Hence the *i*th element of *y* can be interpreted as the probability of the input image being in the *i*th class.

#### 2.1.3. Network optimization   

The parameters of the CNN include the kernels used in the convolution layers and the weights associated with the edges of the fully connected layer. These parameters are ‘learned’ to minimize an overall loss function *L*, defined in terms of the discrepancy between the network prediction and the ground-truth labels of the input images in the training set. This function has the form
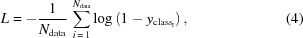
where 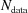 denotes the number of training data, 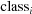 represents the target class of the *i*th data, and 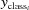 is the predicted probability of target class *i*.

The simplest minimization algorithm for such a highly nonlinear function is the gradient descent algorithm, where at each step of iteration the loss function is reduced along the (negative) gradient direction – the steepest local descent direction. The gradient of the loss function is computed using the chain rule, commonly known as the *back-propagation* algorithm in the neural network literature.

To train a CNN in a supervised learning setting, we split the annotated data into training, validation and testing sets, with the training data used for optimizing the network parameters, and the validation set for tuning hyper-parameters such as the learning rate, or step size along the steepest descent direction in a gradient descent algorithm. The test set is used to demonstrate the accuracy of the optimized network.

Due to the limitation of memory and computational power, it is impractical to evaluate the loss function on the whole training set at once. Instead, we update the learnable parameters iteratively over a mini-batch, a subset created by randomly sampling the images. The parameters are updated using gradient descent over each mini-batch. The updating formula can be expressed as 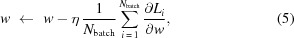
where *w* denotes a learnable parameter, η is the learning rate and 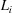 represents the loss function of the *i*th data in the batch. One *epoch* of training refers to the network going through the entire dataset once after using several mini-batches to update the parameter *w*.

To ensure that images of a majority class do not dominate network optimization during the training process, we make sure that all mini-batches have approximately equal numbers of images per class.

When the training set is too small, the model can be trained to fit the training data very well, but it performs poorly on test samples, commonly known as overfitting. Overfitting can be mitigated by using data augmentation techniques to enlarge the training dataset. We describe data augmentation in §2.2.

### 2.2. Data preprocessing   

In this section, we discuss a number of data preprocessing techniques we use to improve the fidelity of the trained CNN model, reduce the training cost, and enhance the desirable features of the raw data that we would like the CNN to recognize.

Data augmentation is a commonly used technique in deep learning to increase the variety of annotated data. By flipping, cropping, resizing and rotating raw images, and scaling their pixel values, we generate additional images that can be used for training. Such an augmentation procedure can reduce overfitting from a limited amount of training data.

**Figure 3:**
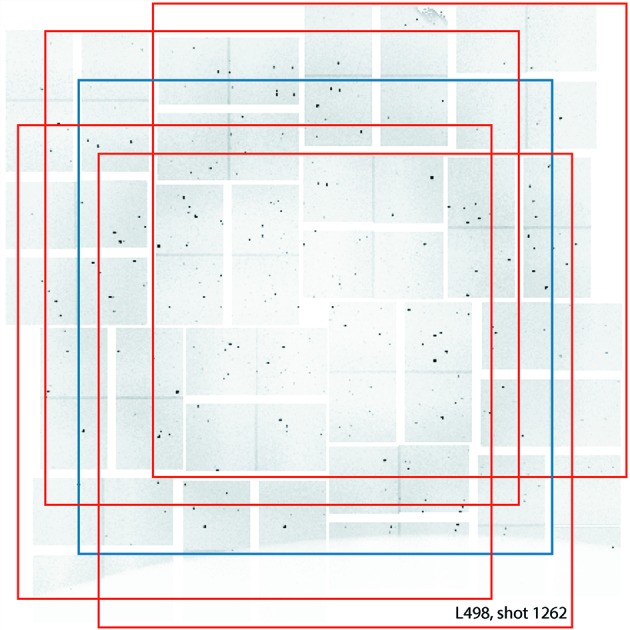
An example of center cropping (blue box) to reduce the image size, and random cropping (red boxes) to generate additional images to augment the training dataset.

Images recorded by modern detectors used in X-ray crystallography experiments typically contain millions of pixels. Using raw images of this size directly to train the CNN can be computationally expensive in both memory usage and CPU time. To reduce the computational cost, we downsample each experimental image by a factor of four (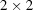 binning). Since Bragg spots are typically located near the center of an image (low-*q* regions), we crop out a 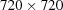 subimage centered at 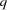 = 0 from each downsampled image to further reduce its size. This cropping process is referred to as *center cropping*. We use a subset of these center cropped images for testing the accuracy of the CNN. In addition to center cropped images, we generate more training images by shifting a downsampled image by a few pixels (uniformly chosen from the range 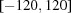) in either the horizontal or vertical direction, prior to center cropping. We refer to this type of cropping as *random cropping*. Fig. 3 ▸ shows how center cropping and random cropping are performed for a specific X-ray image.

The use of random cropping augments the original dataset by introducing spatially translated versions of the input image. It reduces the likelihood of a network overfitting the training set and tends to improve classification performance during testing.

**Figure 4:**
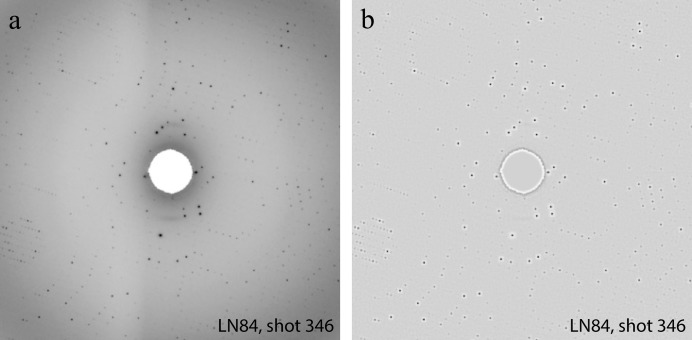
The effects of contrast adjustment through data preprocessing. (*a*) Simple linear contrast adjustment by the program *dials.image_viewer*. (*b*) Image preproccessing by LCN.

In addition, we consider a *local contrast normalization* (LCN) procedure to preprocess the raw image (Jarrett *et al.*, 2009 ▸), since it is sometimes difficult to identify Bragg spots directly from the raw image intensity due to the large dynamic range of pixel intensities recorded by modern detectors and the high intensity of pixels that represent undesirable artifacts. Our implementation is slightly different from Jarrett’s version (Jarrett *et al.*, 2009 ▸) since we first perform a global normalization of the form 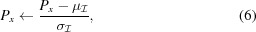
where 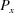 denotes the intensity at pixel *x*, and 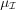 and 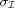 are the mean and standard deviation of the entire image. The globally normalized image is then subject to a local normalization of the form 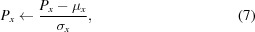
where
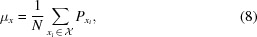


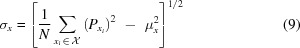
are the local mean and standard deviation at *x*, 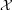 is the set of neighboring pixels of *x*, and *N* is the size of 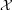. The local normalization has the effect of enhancing the intensity separation of pixels with higher intensities from those of their neighboring pixels. The LCN procedure is designed to reduce the background noise and enhance the contrast between background and Bragg spots. Fig. 4 ▸ shows the effect of applying LCN to a typical image acquired in an X-ray crystallography experiment, and compares the LCN preprocessed image with the same image rendered with the simple linear contrast adjustment used by the program *dials.image_viewer* (described below). As shown in Fig. 4(*b*) ▸, LCN tends to smooth out the variation of the background intensity. While the white halos surrounding the Bragg spots may be visually striking, the importance of LCN for training a CNN classifier lies in its ability to reduce the range of intensities, thus making weight optimization easier and convergence faster.

## 3. Results and discussion   

This section describes the effectiveness of our CNNs to screen images produced from X-ray crystallography experiments.

### 3.1. Datasets   

Protein serial crystallography diffraction datasets used for the Bragg spot analysis are listed in Table 1 ▸, and were collected at the Coherent X-ray Imaging [CXI (Liang *et al.*, 2015 ▸)] and Macromolecular Femtosecond Crystallography [MFX (Boutet *et al.*, 2016 ▸)] instruments of the Linac Coherent Light Source (White *et al.*, 2015 ▸). These experiments were selected as a representative cross section of available imaging detectors, beam energies and sample delivery methods, and inclusive of crystals with different space groups and unit-cell parameters.

CXI data were recorded in vacuum, on a CSPAD pixel array detector (Hart *et al.*, 2012 ▸; Herrmann *et al.*, 2015 ▸; Blaj *et al.*, 2015 ▸), which converts diffracted X-rays into detected charge, directly within a pixelated silicon sensor. In contrast, MFX data were recorded at 1 atm, on a Rayonix MX170HS that employs a coupled system, where X-ray photons are first converted within a phosphor layer into visible light, which is then transmitted through a fiber optic taper to a detecting CCD. As a consequence, there is an appreciable point-spread function such that Bragg spots, which typically cover less than ten pixels on the CSPAD detector, may extend to many tens of pixels in area on a Rayonix detector (Holton *et al.*, 2012 ▸).

Due to this differing appearance of Bragg spots across detector types, it is of interest to assess whether neural networks can be cross-trained to recognize Bragg-spot-containing patterns from both instruments.

**Figure 5:**
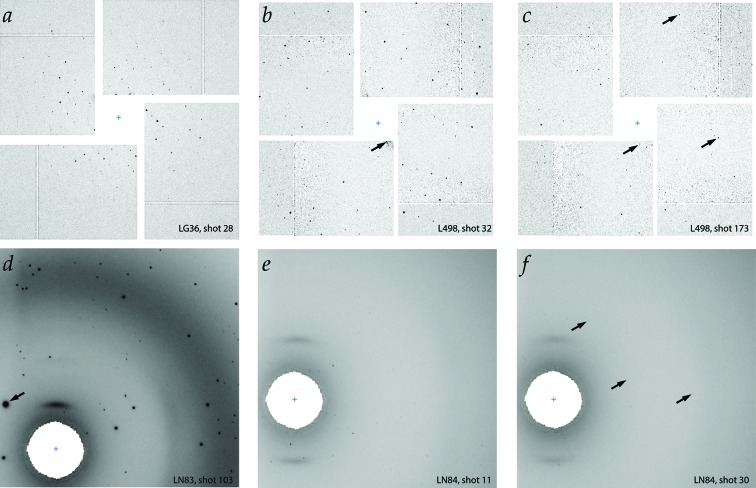
Challenges in expert hit recognition. (*a*) The large unit cell of photosystem II presents a clear, densely spaced lattice with CSPAD detection. (*b*, *c*) The smaller unit cell and high crystalline order of thermolysin generate a more difficult scenario. Some images (*b*) show an easily recognized lattice, while others (*c*) require careful inspection. Several Bragg spots in (*c*) have only a single pixel square area, and can only be recognized by contrast optimization, blink comparison against neighboring images and evaluation of the spot density, which is greatest at low scattering angle. (*d*, *e*, *f*) Rayonix images with a large point spread function often display easily recognized spots; however, weak spots are harder to recognize because they lack contrast with the background. Arrows indicate (*b*) a diffraction spike due to the liquid jet sample-delivery system, (*c*) thermolysin Bragg spots 2–3 pixels in square area, that are oriented radially with respect to the direct beam, (*d*) a hydrogenase spot with area nearly 400 pixels in square area, due to large spot intensity and point spread function, and (*f*) photosystem II Bragg spots with very low contrast against the background.

**Figure 6:**
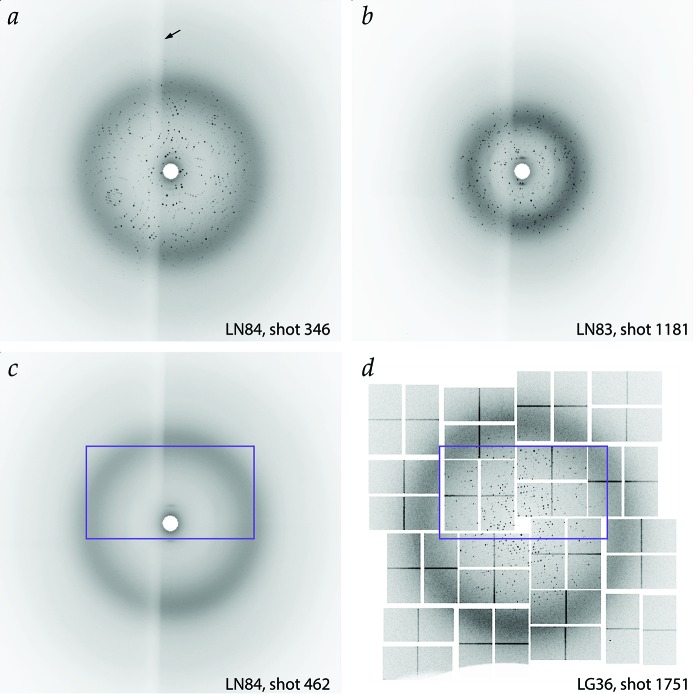
Representative diffraction patterns from the Rayonix (*a*, *b*, *c*) and CSPAD (*d*) detectors. The photosystem II lattice patterns (*a*, *d*) illustrate diffraction from single crystals that are readily indexed, while the pattern from hydrogenase (*b*) contains many overlapping lattices (several crystals are in the beam) that are relatively difficult to disentangle. Pattern (*c*) is a ‘Miss,’ containing solvent diffraction only, no protein. The purple boxes (*c*, *d*) show the approximate inspection area for the human expert annotation. The figure illustration was produced by binning the original data 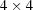. Gray-scale display values *D* rendered in the viewer in the range [0 = white, 1 = black] were calculated as follows: *D* = min(1, MP_*x*_/P_90_), where 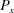 is the value at pixel *x* in the raw data, 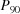 is the value of the 90th-percentile pixel over the image, and *M* is a contrast parameter set manually within the viewer, with values in the range [0.004 = lightest, 2 = darkest], with a default of 0.4. The arrow in (*a*) highlights the shadow cast by partial absorption of X-rays through the Kapton material of the conveyor belt.

Several distinct protocols were used to deliver the crystal stream to the X-ray beam, each of which contributes unique artifacts to the resulting images. Crystal specimens at the CXI instrument were delivered with a liquid jet focused by an electric potential, in either the microfluidic electrokinetic sample holder (MESH) configuration (Sierra *et al.*, 2012 ▸) for L498 or the concentric MESH (coMESH) configuration (Sierra *et al.*, 2016 ▸) for LG36, either of which can generate occasional diffraction spikes from the liquid jet at low diffraction angles (Fig. 5 ▸). Experiments at MFX were conducted with conveyor-belt delivery of crystal specimens (Fuller *et al.*, 2017 ▸) for LN84 and LN83, which partially shadows half of the diffraction pattern that passes through the Kapton belt material (Fig. 6 ▸). For experiment LO19, the crystals were delivered by a liquid jet in the gas dynamic virtual nozzle configuration (Weierstall *et al.*, 2012 ▸).

Finally, the data chosen represent a wide range of unit-cell parameters and therefore Bragg spot densities, covering Bragg spot densities that are sparse for cyclophilin A (Keedy *et al.*, 2015 ▸), intermediate for thermolysin (Hattne *et al.*, 2014 ▸) and O_2_-tolerant [NiFe]-hydrogenase (Fritsch *et al.*, 2011 ▸), and crowded for photosystem II (Young *et al.*, 2016 ▸).

### 3.2. Preparation of the data   

For each experimental run, the first 2000 images were unpacked from the native LCLS data format (XTC, extended tagged container) and converted to a 4-byte integer HDF5 format for further study. For the CSPAD detector, the raw (2-byte) XTC data were recorded individually on 64 application-specific integrated circuits (ASICs), each covering a 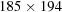 pixel array. For our analysis, these data arrays were re­assembled into their approximate physical locations (to within ∼1 pixel accuracy) within a monolithic 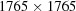 data array, which the incident X-ray beam intersects near the center. Pixels on the monolithic array outside of the 64 active rectangles were set to a pixel value of 0 throughout the analysis. An average dark-current array was subtracted from each image. While this inevitably produced a few pixels with negative values, it did not have an impact on the ability to process the images with a CNN.

For the LG36 data, pixels were individually set during data collection to be in either high- or low-gain mode (differing in the ratio of detector counts to incident photons). This allowed weak Bragg spots outside of the 3.2 Å cone to be accurately recorded in a high-gain mode, while the low-gain setting was employed for bright low-angle Bragg spots, to help avoid detector saturation. Therefore, when producing the final HDF5 array, low-gain pixels were multiplied by a factor of about seven, to place inner and outer zones on an equivalent scale.

Various experimental artifacts may be seen in the CSPAD image data (Fig. 6*d* ▸). The double row of pixels forming the border between ASIC pairs always produces pixel values higher than the surrounding background, because these pixels pick up extra X-ray signal from the inter-ASIC spacing. Additionally, the lower strip of ASICs from experiment LG36 is shadowed by equipment in the vacuum chamber, so the affected area records no X-rays. We should note that the preprocessing methods of §2.2 do not explicitly remove these artifacts. For example, we do not explicitly mask out or remove the edges of the CSPAD detector tiles, or flag the white space in between tiles (represented by pixels set to a value of 0). Although it is intuitively desirable to remove these artifacts, we found that this type of data preprocessing yields little performance improvement for a CNN. A possible explanation is that CNN is trained to ignore such artifacts since they are present regardless of whether the image is a Hit or Miss.

For the Rayonix detector, raw data were binned 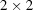 in hardware, giving a final 2-byte data array of 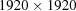 pixels. The data array is of monolithic construction, except for the presence of a large hole in the center to allow passage of the incident beam. These central pixels were set to a value of 0.

For both detectors, it was impossible to avoid saturating the detector at the positions of the brightest Bragg spots, and consequently these pixels are pegged at a high setting within the final images.

### 3.3. Reference annotation   

Two methods were used to provide a reference classification for each image: annotation by a human expert, and the use of an automated spotfinder in conjunction with thresholding. It was necessary to decide at the outset what criteria to use for classification. One possibility is to report images that can be indexed (Fig. 6*a* ▸), where indexing is defined as the successful refinement of a model of the unit-cell parameters and lattice orientation. However, it can be argued that lattice indexing as a criterion to veto events is far too stringent, for much information can be learned about the crystalline sample even if the images cannot be explicitly indexed. For example, the limiting resolution can be determined, some idea of the crystal disorder (mosaicity) can be gained, and it can be assessed whether the images contain multiple lattices that cannot easily be disentangled (Fig. 6*b* ▸). Therefore, we focused exclusively on classifying ‘Hits’ based on whether or not Bragg spots are visible, in contrast to images that are either blank or that contain diffraction from diffuse scattering or water rings (Fig. 6*c* ▸), but not from single crystals.

#### 3.3.1. Expert classification   

HDF5-format data were annotated by inspection within the program *dials.image_viewer*, using specialized plug-ins for image format and pushbutton scoring. The viewer contrast was set manually so that low-angle Bragg spots could be distinguished visually from the background air and solvent scatter; however, within each experiment the images were viewed at a constant level of contrast. For the CSPAD pixel array detector, and in particular for the L498 thermolysin dataset, it was found that many Bragg spots were only 1 pixel in square area (Fig. 5*c* ▸). Therefore, data were viewed at a zoom level where one image pixel was rendered over an area of 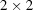 screen pixels. Since the work was done on a 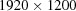-pixel monitor, it was only possible to inspect an approximately 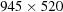-pixel area of the image. As seen in Figs. 6(*c*) and 6(*d*) ▸, the inspection window was positioned with the incident beam located at the lower center of the screen. Images were classified as ‘Hit’ if ten or more features were recognized as Bragg spots, ‘Maybe’ with four to nine Bragg spots, otherwise ‘Miss.’

The classification exercise presented varying levels of difficulty among the five experiments. L498 was the most challenging (Figs. 5*b* and 5*c* ▸). Due to the high degree of crystalline order for thermolysin, and the small point spread function of the CSPAD detector, there are many Bragg spots occupying only one pixel in square area, and these Bragg spots are difficult to distinguish from the noisy background. Spots are sparsely distributed on the images owing to the small unit-cell dimensions, and thus for images with only a few spots there is no discernible lattice pattern. Recognition of hits often relies on the identification of individual Bragg spots in isolation. For those spots extending over several pixels, a diagnostic feature is the elongation along the radial direction, due to the combination of the mosaic structure of the crystal, and the spectral dispersion of the beam as previously described (Hattne *et al.*, 2014 ▸). Also, for evaluating whether features are credible Bragg spots, priority was given to those candidates that cluster at low scattering angles (near the direct beam), yet are uniformly distributed at all azimuthal angles surrounding the beam position. The L498 experiment is unique in having more images classified as ‘Maybe’ than ‘Hit’. Also, it was found that in repeat classification attempts, the Hit/Maybe and Maybe/Miss distinctions are somewhat uncertain for L498.

In contrast, the large unit-cell dimensions of photosystem II produce crowded Bragg spot patterns in the LG36 experiment, where the lattice repeat is often immediately evident, making classification much easier. Due to the spot density, many fewer images are designated as ‘Maybe’. A large number of ‘Miss’ images contain neither Bragg spots nor water rings, indicating that the liquid jet has missed the beam.

The LN84 data likewise contain photosystem II diffraction (Figs. 5*e* and 5*f* ▸) but, due to the significant point spread function of the Rayonix detector, Bragg spot candidates are spread (isotropically) over many pixels, and often cover 10–30 pixels in square area. For those images that contain only a few weak Bragg spots at low scattering angle, it is more difficult than with the CSPAD to recognize these as Hits or Maybes, as the signal presents less contrast with the background (Fig. 5*f* ▸). Care had to be taken to set the contrast within the viewer display so as to increase the prominence of these weak spots. Within the LN84 dataset, it is observed that many of the patterns contain overlapping lattices (multiple crystals) and that layer lines (rows of Bragg spots) are sometimes smeared out.

The LN83 (hydrogenase; Fig. 5*d* ▸) and LO19 (cyclophilin A) datasets do not show smearing within the layer lines, but do exhibit multiple overlapping lattices to some extent.

#### 3.3.2. Automatic spotfinding   

Several open-source software packages are available for automatic hit-finding performed on XFEL data, including *Cheetah* (Barty *et al.*, 2014 ▸) and *cctbx.xfel* (Sauter *et al.*, 2013 ▸). Within the *cctbx.xfel* package, automatic spot picking algorithms used for hit-finding are implemented within both the *CCTBX* (Zhang *et al.*, 2006 ▸) and *DIALS* (Winter *et al.*, 2018 ▸) component toolboxes. For the present project, which employed the *DIALS* implementation, we found it necessary to carefully tune the spotfinding parameters (Lyubimov *et al.*, 2016 ▸) for each experiment when counting the Bragg spot candidates for automatic annotation. Prior to the spotfinding process, each experiment was evaluated to determine the set of ‘untrusted’ pixels to be ignored during annotation. For CSPAD images, the untrusted pixels included those pixels found to be ‘cold’ or ‘hot’ during a dark run, those known to lack bump-bonding to any detector circuit, and those on the 1 pixel-wide rectangular perimeter of any ASIC. For both detector types, the image viewer was also used to outline a set of pixels for each experiment that are completely shadowed from X-rays.

**Figure 7:**
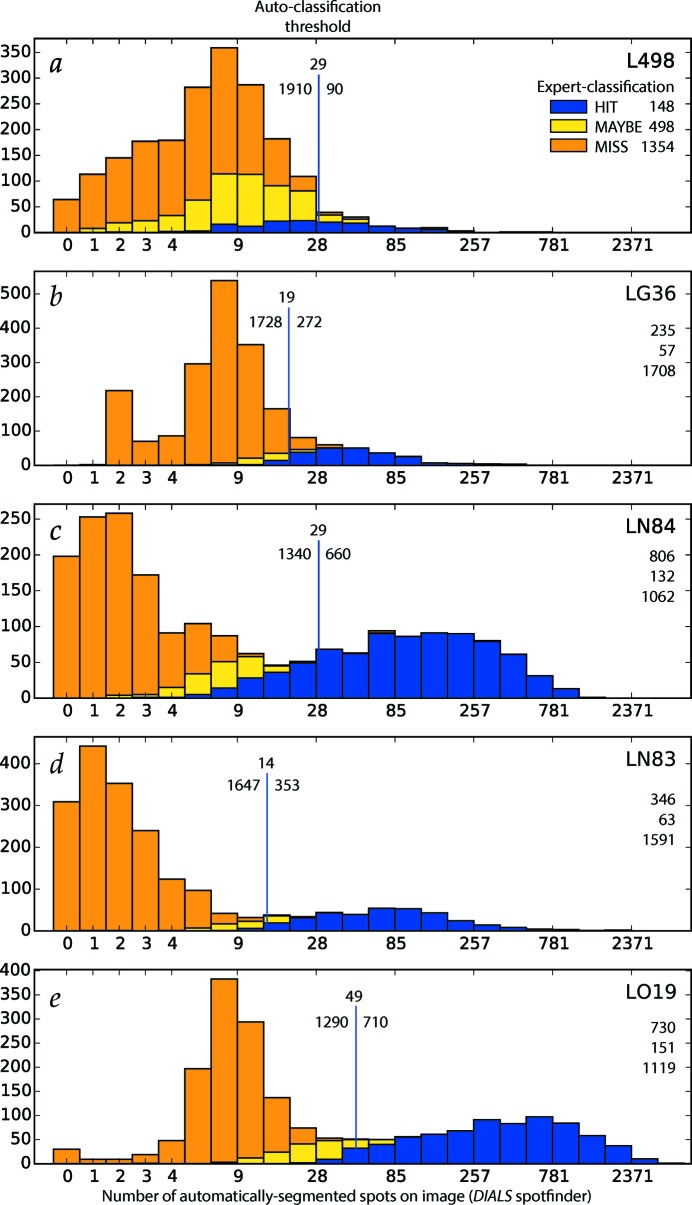
Comparison of expert and automatic image classification trials for experiments L498 (*a*), LG36 (*b*), LN84 (*c*), LN83 (*d*) and LO19 (*e*). From the 2000-image source data, frames were divided into ‘Hit’, ‘Maybe’, ‘Miss’ classes by a human expert, with class totals listed at the upper right of each panel. Separately, candidate Bragg spots were automatically segmented on each image by the *DIALS* spotfinder, thus producing a histogram of the number of Bragg candidates per image, with the number of spots plotted here on a logarithmic scale over the horizontal axis. A best-guess threshold for classifying images as hits or misses is indicated for each experiment as a vertical line, along with the number of images falling on each side of the threshold. The correspondence between expert and automatic classifications is indicated by blue, orange and yellow shading on the bar graphs.

Tunable parameters chosen for each experiment are listed in Table 2 ▸, and were optimized by inspecting the results of automatic spot segmentation within the image viewer. Furthermore, an attempt was made, for each experiment, to determine a threshold count of automatically found Bragg spot candidates, above which the image is classified as a ‘Hit’. Best-attempt threshold values from visual inspection are shown in Fig. 7 ▸ (vertical blue lines). Table 2 ▸ lists the wall clock time required to annotate 2000 images from each experiment with *dials.find_spots* using a 16-core, 64-bit Intel Xeon E5-2620 v4 processor (2.1 GHz) with 20 MB cache, 128 GB RAM, running Red Hat Enterprise Linux Server 7.3. C++ code was compiled under GCC 4.8.5, and a Python wrapper provided 16-core multiprocessing for load balancing.

Fig. 7 ▸ and Table 3 ▸ show that the automatic classification results are only moderately successful, when compared with the ‘ground truth’ of expert classification. For the three Rayonix experiments (LN84, LN83 and LO19) it was possible to identify good threshold values above which all hits are expert-classified as ‘Hit’ or ‘Maybe’, although for LN84 a significant fraction of ‘Hit’ images fall below the automatic threshold. The outcome is worse for the CSPAD data sets, where the chosen threshold leads to the false-positive classification of several ‘Miss’ images. The cause turns out to be the grainy structure of the water ring, as detected on the CSPAD, which cannot be distinguished from true Bragg spots using the simple criteria used here. Finally, for L498, about half of the ‘Hit’s fall below the automatic threshold.

In view of these results, all CNN training described below is performed with the expert annotation.

### 3.4. Experiment-specific training and testing   

LCN preprocessing was performed on a 16-core, 64-bit Intel Xeon E5-2620 v4 server (consisting of two 8-core chips, 2.1 GHz) with 20 MB cache, 128 GB RAM, running Ubuntu Linux Server 16.06, with code programmed in C, utilizing an algorithm implemented in the Torch library^3^ compiled under GCC 5.4.0. LCN preprocessing, which was the rate-limiting step for image classification, required 5.7 s per batch of 64 images, using parallel execution on 8 CPU cores of the Linux server.

CNN networks were implemented with the Torch framework (http://torch.ch), and were trained and tested using an Nvidia 1080Ti GPU extension card (1.58 GHz) plugged into the Ubuntu server, with 11.2 GB on-device memory, 2048 threads per multiprocessor, and 32-bit precision operations programmed in CUDA. GPU operation was controlled by an Ubuntu thread operating in parallel to the LCN preprocessing step. Training required 0.26 s wall clock time (for the total of 120 epochs) per batch of 64 images, and testing required 0.05 s per batch. Therefore, the CNN processing operations depicted in Fig. 2 ▸ were run at a considerably higher throughput rate (1280 Hz) than the current framing rate (120 Hz) at LCLS.

For each dataset listed in Table 1 ▸, we separately train a CNN using 50% randomly selected images as the training data, 20% of the images as the validation data, with the accuracy of the trained network being tested on the remaining 30% of the images.

We use the same set of hyperparameters to train the network in all the experiments. Each mini-batch has 64 images, and each epoch contains 100 batches. We train the network for 120 epochs. We initialize the learning rate to 0.1 and allow it to exponentially decrease to 0.0001.

**Figure 8:**
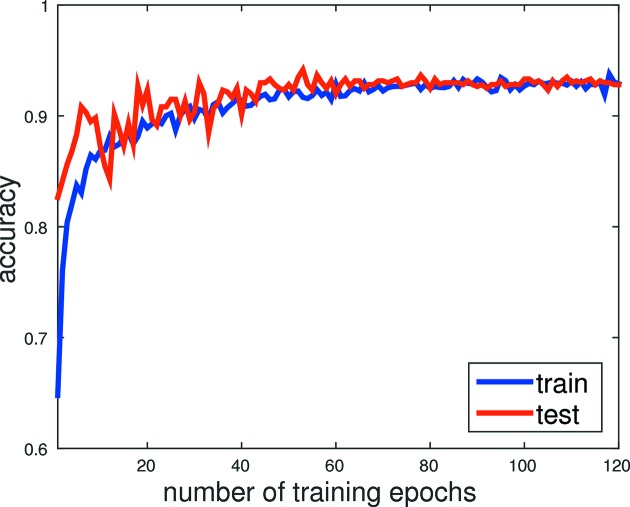
Training and testing accuracy over 120 epochs for the LO19 dataset. The test accuracy was never used for optimizing the CNN model during training.

Fig. 8 ▸ shows the training history of the CNN for the LO19 dataset. We plot the accuracy of the prediction, measured in terms of the percentage of successful predictions on both the training (blue curve) and testing (red curve) image data, over the number of epochs. We observe that after being trained for 10 epochs the CNN exhibits over 90% success rate in predicting whether a training image is a ‘Hit’, ‘Maybe’ or ‘Miss’. The success rate continues to improve until it reaches around 93%.

Table 3 ▸ shows the *confusion* matrices associated with the CNN classifications, compared with the ground truth annotation by the human expert. We also compare the automated spotfinding software against the ground truth.

The confusion matrix measures the accuracy of a classification method. Its rows represent the ground-truth (expert-annotated) classes of the data, and its columns represent the predicted class, either from CNN or automatic spotfinding. The *i*th diagonal entry of the matrix represents the percentage of class *i* images that have been correctly predicted as class *i*. The (*i*,*j*)th off-diagonal entry represents the percentage of class *i* images that are misclassified as class *j* images.

To compare the accuracy of the CNN prediction, which produces three different classes (Hit, Maybe and Miss), directly with that of the spotfinder prediction, which returns one of the two classes (Hit and Miss) based on a hard threshold of Bragg spot counts, we group the Hit and Maybe classes predicted by the CNN together. The (1,1)th diagonal entry records the percentage of images that are correctly predicted to be either a Hit or a Maybe, and the (1,2)th off-diagonal entry records the percentage of images that are labeled as Hit or Maybe, but predicted to be Miss.

We can see from Table 3 ▸ that, for all datasets, over 90% of the images that have been classified by a human expert as ‘Miss’ have been correctly predicted by the CNN. Although these percentage numbers are slightly lower than those produced by the spotfinder, which range from 98% to 100%, they are already quite high, and indicate that CNN can be reliably used to exclude images that do not capture crystal diffraction events from being stored. A small percentage of false positives that are retained can be identified later through other image analysis tools.

Table 3 ▸ indicates that CNN does a much better job than automatic spotfinding in identifying images that a human expert considers to be a Hit or a Maybe. This is particularly true for difficult datasets such as the L498 dataset in which Hit and Maybe images contain a relatively small number of Bragg spots that occupy only one pixel square area. To some extent, this is not entirely surprising, because the CNN is capable of encoding human vision and knowledge into the network parameters so that it can make predictions more like a human expert.

**Figure 9:**
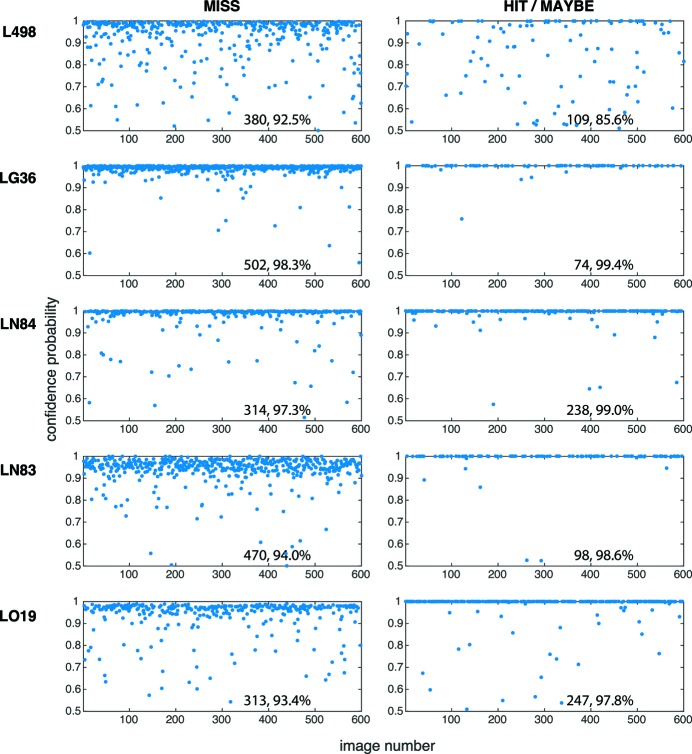
Reliability of the test data set classification, as shown by the confidence level represented by the CNN classification vector, for CNNs trained on data from the same LCLS run. Within each test data set of 600 images the left column shows classification probabilities for images correctly classified as Miss, while the right column shows the sum of the Hit + Maybe probabilities for those images correctly classified as either Hit or Maybe. Each panel gives the total number of correct classifications, and the average confidence probability for those images correctly classified. Notably, the L498 network, that has a low success rate for predicting Hit/Maybe (Table 3 ▸), also has a low confidence level in Hit/Maybe classification. In contrast, for networks such as LN83, the probabilities associated with most Hit/Maybe images are above 90%, indicating that these images are relatively easier to classify.

Although we are ultimately interested in whether each X-ray diffraction image should be considered as a Hit or Miss, it is also useful to know how much confidence we should place on the classification given by a CNN. This information is provided by the classification vector produced by the SoftMax layer of the CNN. The *i*th component of the vector gives the estimated probability that the image to be classified is in the *i*th class. Fig. 9 ▸ shows the estimated class probabilities associated with all correctly classified images. The left column shows the estimated probabilities of Miss associated with images that are correctly predicted to be Miss, while the right column shows the aggregated probabilities of Hit and Maybe associated with images that are correctly predicted to be either Hit or Maybe. The aggregated probability is obtained by simply adding the probability of an image predicted to be a Hit with the probability of it being a Maybe. We observe from this figure that, with the exception of the L498 dataset, the confidence level of most correctly classified (both Miss and Hit/Maybe) images are relatively high with estimated probabilities above 90%. The difficulty associated with the L498 dataset is revealed from the estimated class probabilities associated with images that are correctly predicted to be Hit or Maybe.^4^

**Figure 10:**
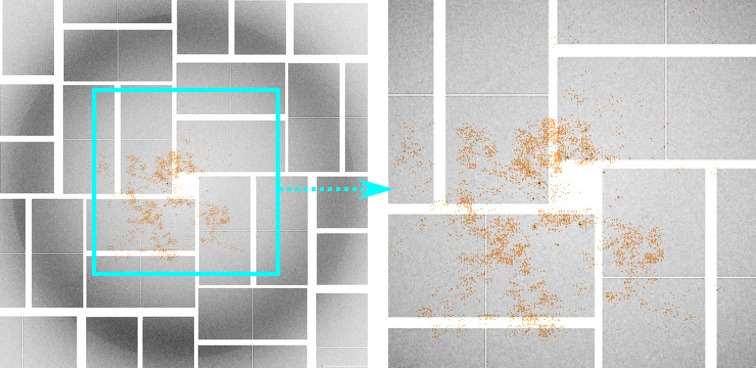
The magnitude of the gradient of the loss function *L* with respect to the pixel intensity for an LG36 input image overlayed on the input image. Only the largest magnitudes relative to the mean of the gradient components are shown as the brown shaded pixels, as they are most responsible for the model prediction. Magnitudes smaller than three standard deviations below the mean are disregarded.

One remarkable capability of the CNN is that it can learn to ignore most of the noise and undesirable artifacts even though the nature of these factors is not explicitly characterized in the training process. Once trained, the CNN tends to focus on areas of the input image that are likely to contain important features pertinent to classification. To visualize this effect, we plot in Fig. 10 ▸ the absolute value of the gradient of the loss function *L* defined in equation (4) with respect to the pixel intensities *x* of an LG36 input image. The gradient is evaluated at optimized CNN parameters obtained from the training process. Each element of the gradient 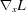 indicates how sensitive the loss function is to the change of the corresponding pixel. Since 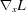 is generally nonzero for all pixels, we want to visualize only those with the highest and lowest gradient values relative to the mean (*i.e.* the most sensitive pixels seen by the CNN). Fig. 10 ▸ plots elements of 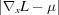 whose values are 3σ above or below the mean μ of 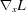, where σ is the standard deviation of 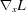 over all pixels. The pattern suggests that the most sensitive pixels do indeed cover the general region of the image where Bragg spots can be found. It is important to note that the most sensitive pixels cover not just the Bragg spots but also some adjacent areas, thus apparently helping the CNN recognize the presence of Bragg diffraction against background. Areas of the image that contain unimportant artifacts such as the water ring appear to be excluded.

**Figure 11:**
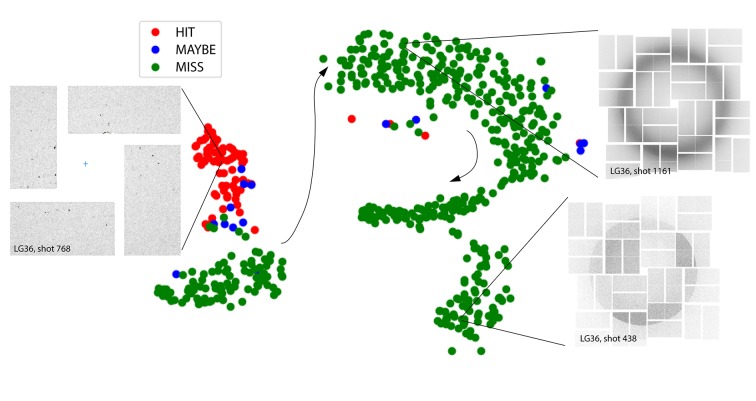
Stochastic neighbor embedding of the feature maps extracted from an LG36-trained CNN applied to the LG36 dataset. Each spot on the map corresponds to one image, and is colored by its ground-truth classification of Hit (red), Maybe (blue) or Miss (green). On such an embedded map the *x,  y* coordinates have no specific physical interpretation; indeed, the final coordinates of the spots are dependent on the random number seed utilized. Rather, the utility of the plot lies in the fact that near neighbors within it display similar image characteristics, as encoded by their feature vectors. A visual inspection of the images then reveals what the similarities are. Image insets illustrate the cluster of Bragg-spot hits exemplified by shot 768, as well as the lower-right cluster, exemplified by shot 438, which turns out to contain no diffracted photons (no X-ray beam, dark noise only). Miss images that contain only a water ring, exemplified by shot 1161, form a continuous distribution, shown by the black arrows, with the arrows’ arc roughly indicating an ordering from strongest to weakest water signal.

Although it is well understood that a CNN makes a prediction by extracting important features of an image through a multi-layer convolution / batch normalization / ReLU / maxpooling process, it is generally difficult to interpret the resulting feature maps directly by human vision. However, these feature maps are believed to be more classifiable than the original image stack. In a CNN, the final classification is performed by the last layer of the network that connects every feature map to every categorical probabilistic distribution neuron (*i.e.* the fully connected layer), yet there are other nonlinear classification techniques that can potentially yield more information about the feature maps. One such technique is based on the T-distributed stochastic neighbor embedding (t-SNE) (van der Maaten & Hinton, 2008 ▸) method, a nonlinear dimensional reduction technique that can be used to preserve high-dimensional structures at different scales. The t-SNE method embeds the feature vectors into a two-dimensional plane that can be easily visualized, while preserving nearest neighbor relationships between feature vectors from similar images. Fig. 11 ▸ shows the t-SNE of a set of 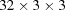 feature maps extracted from the last layer of the LG36-trained CNN, with each dot in the plot corresponding to one of the LG36 test images. As we can see, images that are considered to be Miss (green) by a human expert are clearly separated from the images that are considered to be Hit (red). Images that are considered to be Maybe (blue) lie somewhat in between the Miss and Hit clusters. Therefore, this figure serves to confirm that the LG36-trained CNN produces feature maps that can be easily clustered when it is applied to test images in the LG36 dataset. It is also interesting that t-SNE actually reveals additional feature clusters (such as blank images) that are not explicitly annotated in the training process.

### 3.5. Cross-dataset training and testing   

We envision the eventual application of CNNs as a screening tool in serial X-ray crystallography experiments, in which standard diffraction data are expert-annotated for the training process, after which the CNN can be deployed for real-time classification of new datasets from experiments that presumably employ similar sample delivery systems and detectors as those used for the training data. The results presented above are encouraging, but do not directly address the question of how similar the new experiment will have to be to the previous experiments used for annotation and training.

In this section, we investigate to what extent a CNN trained on one data set can be applied to data acquired under differing conditions. Table 4 ▸ lists the success rates achieved when a CNN trained with one Rayonix dataset is applied to the other Rayonix datasets, where success rate is defined as 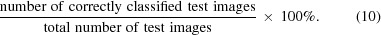
A high success rate is mutually obtained by cross-application between LN84 and LN83, both of which examine large-unit-cell protein crystals delivered by conveyor belt. However, the LO19 trained CNN yields relatively lower success rates when applied to both the LN84 and LN83 test images. We suspect that the low success rate is due to the difference in the sample delivery method used in LO19 (GDVN) in contrast to the LN84/LN83 experiments.

Table 5 ▸ shows that a CNN trained with the combined group of experiments detected with the Rayonix device (LN84, LN83 and LO19) yields a relatively high success rate (92%) when applied to test images within the Rayonix group. However, when applied to the LG36 or L498 CSPAD datasets, the success rate is rather low, most likely due to the differing tile geometry of the CSPAD detector (Fig. 6*c* *versus* Fig. 6*d* ▸) that the Rayonix-trained CNN does not know how to characterize. Table 6 ▸ shows that by combining the LG36 training data with that of the Rayonix dataset we were able to train the CNN to perform reasonably well on testing images in both the Rayonix and LG36 datasets. However, the new CNN still performs rather poorly (with a 54% success rate) on the testing images in the L498 dataset. The performance of the CNN improves slightly (to 74% success rate) after we add images from the L498 dataset to the training data and retrain the CNN from scratch.

### 3.6. Prediction accuracy *versus* data size   

To reduce the amount of work done by a human expert to annotate experimental images used for CNN training, we would like to keep the training dataset as small as possible. Table 7 ▸ shows that we can achieve over 91% success rate in screening the Rayonix (LO19, LN83, LN84) and LG36 test images when as few as 100 images selected from the combined Rayonix and LG36 datasets are used to train a CNN. As we increase the number of training images, we achieve a slightly higher success rate for the Rayonix testing images (94% when 4000 training images from the combined Rayonix and LG36 datasets are used). However, increasing the size of the training set does not seem to improve the success rate for LG36 consistently. In fact, the success rate goes from 95% for a CNN trained with 100 combined Rayonix and LG36 images to 91% for a CNN trained with 4000 images. Nonetheless, the overall success rate is still acceptable. Therefore, when a new CNN is constructed and trained for a new experiment, the number of images required to be examined and annotated by a human expert is of the order of a few hundred, which is more manageable. Annotating more images can possibly improve the success rate, but the level of improvement is likely to be problem dependent.

### 3.7. The effect of data preprocessing   

We found that it is important to preprocess the data using the LCN technique discussed in §2.2. Without any contrast adjustment, it is almost impossible to train a CNN to make an accurate prediction (Table 8 ▸). We believe that, when supplied with the raw data, CNN training is dominated by artifacts and background, while the LCN process visibly removes these features, so that training can focus on Bragg spots. Table 8 ▸ shows that this approach appears to be more effective than the simple linear contrast adjustment employed by *dials.image_viewer*.

## 4. Conclusion   

In this paper, we investigated the feasibility of using pre-trained CNNs as a screening tool to veto certain diffraction events, *e.g.* images that contain no Bragg spots, in a serial X-ray crystallography experiment, so that data with little value can be quickly pruned and discarded instead of being stored for further examination and analysis. The screening process selects images considered to be Hits, so they can be indexed, integrated and merged into the downstream data processing pipeline for biological structure determination. We showed that with a modest set of carefully annotated images collected from previous experiments, a CNN can be trained to successfully distinguish Hits from Misses in most cases.

The major benefit of a CNN is that it does not require a precise characterization of the Bragg spots or of the many undesirable artifacts present in the images. Indeed, artifacts are often difficult to quantify or describe mathematically. Other than using local contrast enhancement to improve image contrast and smooth out the background, and augmenting the dataset by random cropping, we do not use any sophisticated image processing techniques to isolate Bragg peaks from noise or other types of random or systematic artifacts. Compared with automatic spotfinding tools implemented in the *DIALS* software toolbox, which still require manual parameter tuning, the CNNs trained here have a slightly lower success rates, but still above 90%, in identifying images that are considered to be Misses by a human expert. However, they have much higher success rates in identifying images that are considered to be Hits.

The CNN implementation showed the ability to identify key diffraction features (the presence of Bragg spots) *via* a learning process that extracts relevant features from the raw image processed through a neural network that consists of convolution / batch normalization / rectification / downsampling layers. These features can then be used to separate data into distinct classes (in this case, Hit, Miss and Maybe) through a so-called fully connected neural layer. The confidence level of classification may be ascertained from the class probability vectors produced by the final SoftMax layer. Although we are primarily interested in whether the images represent Hits or Misses, we found that stochastic neighbor embedding, based on the final feature vector, can suggest more granular classification categories, or reveal unanticipated structure in the data or experiment settings. Fig. 11 ▸, for example, shows that the Misses containing solvent only (no crystals) can be ordered by the intensity of the water ring signal, and that a separate category of Misses can be identified having no X-ray pulse intensity.

We found that the accuracy of CNN classification depends largely on the quality of the annotated data used to train the network. When the training dataset contains a limited number of images with clearly identifiable Bragg spots (such as our L498 dataset), and/or when the number of identifiable Bragg spots within the images is relatively small, the CNN trained by such a dataset tends to have lower inference accuracy when applied to testing data. Because images produced by serial X-ray crystallography reflect the type of detector used, beam properties such as energy, dispersion and divergence, and the sample preparation and delivery method (none of which are part of the annotation used for training), a CNN trained on one dataset cannot necessarily be applied to images collected with different experimental methods. Although it is desirable to train a universal CNN that can be used to screen images from all experiments, the present results suggest that training such a CNN may be difficult, or may require larger datasets than those used here. A reasonable compromise is for CNNs to be customized for the detector and sample delivery techniques at particular X-ray endstations.

With serial crystallography framing rates of 1–100 kHz now and in the next ten years, managing the high data flow rate will continue to be a challenging aspect of experimental design. While it is presently possible to archive all images for future inspection, and marginally possible to process most data for immediate feedback, it is expected that at some future time it will be necessary to throw away some fraction of the data at the time of collection, in order to enable faster data acquisition rates. CNN classification based on the presence of Bragg spots offers one possible avenue to quickly sort the data into productive and unproductive piles, thus economizing on data storage and network bandwidth. We have shown that the CNN classification step can easily be run on a single GPU expansion card at throughput rates (1.3 kHz) that are consistent with current detectors. While our data processing pipeline also included a LCN preprocessing step running at slower throughput rates when implemented on a Linux server, this does not pose a fundamental barrier, as this step could also be easily ported to the GPU platform. A deeper problem, requiring further research, is whether the classification process can be implemented and/or accelerated in an energy-efficient manner by co-locating it with the detector, possibly on hardware that is specialized to implement CNNs. Candidate architectures include neuromorphic chips [known for their low energy consumption (Merolla *et al.*, 2014 ▸) and thus suitable for embedded systems], or field programmable gate arrays, which are becoming roughly comparable with GPUs in both energy consumption and computing throughput (Nurvitadhi, 2017 ▸).

## 5. Data and code   

Diffraction images have been deposited with the Coherent X-ray Imaging Data Bank (Maia, 2012 ▸), accession number 76, at http://cxidb.org/id-76.html. The software package *DIALS* may be downloaded from https://dials.github.io. Scripts used for expert and automatic annotation, LCN preprocessing and CNN classification are available at https://github.com/nksauter/fv5080.

## Figures and Tables

**Table 1:** Experimental data

LCLS dataset (proposal, run)	Incident energy (keV)	Protein	Space group, unit cell (Å)	Instrument	Sample delivery system	Detector
L498, 27	9773	Thermolysin	*P*6_1_22, *a* = 93, *c* = 130	CXI	MESH	CSPAD
LG36, 87	7088	Photosystem II	*P*2_1_2_1_2_1_, *a* = 118, *b* = 224, *c* = 331	CXI	CoMESH	CSPAD
LN84, 95	9516	Photosystem II	*P*2_1_2_1_2_1_, *a* = 118, *b* = 223, *c* = 311	MFX	Conveyor belt	Rayonix
LN83, 18	9498	Hydrogenase	*P*2_1_2_1_2_1_, *a* = 73, *b* = 96, *c* = 119	MFX	Conveyor belt	Rayonix
LO19, 20	9442	Cyclophilin A	*P*2_1_2_1_2_1_, *a* = 42, *b* = 52, *c* = 88	MFX	Liquid jet	Rayonix

**Table 2:** Automatic spotfinding parameters

Experiment	Gain (ADU photon^−1^)	Global threshold (ADU)	Sigma strong	Minimum spot area (pixels)	Wall clock time (s), 16 processors
L498	4.55	100	6	3	100.2
LG36	15.64	100	3	2	79.3
LN84	0.31	200	3	3	89.2
LN83	0.27	200	3	3	80.3
LO19	0.19	200	3	3	80.9

**Table 3:** Confusion matrices for classification of the test data

		CNN		Automatic spotfinding	
Dataset	Human expert	Hit or Maybe	Miss	Hit	Miss
L498	Hit or Maybe	69.0%	31.0%	12.8%	87.2%
	Miss	6.9%	93.1%	0.5%	99.5%
LG36	Hit or Maybe	91.1%	8.9%	77.7%	22.3%
	Miss	1.8%	98.2%	2.6%	97.4%
LN84	Hit or Maybe	98.5%	1.5%	69.9%	30.1%
	Miss	10.1%	89.9%	0.4%	99.6%
LN83	Hit or Maybe	98.5%	1.5%	85.8%	14.2%
	Miss	3.1%	96.9%	0.1%	99.9%
LO19	Hit or Maybe	94.8%	5.2%	80.6%	19.4%
	Miss	4.0%	96.0%	0.0%	100.0%

**Table 4:** Screening success rate of applying a CNN trained with one Rayonix dataset to another Rayonix dataset

Train/test	LO19	LN83	LN84
LO19	93%	85%	65%
LN83	91%	96%	90%
LN84	74%	92%	90%

**Table 5:** Success rate of using a CNN trained with a Rayonix, LG36 or L498 dataset to screen images contained in the same or a different dataset

Train/test	Rayonix	LG36	L498
Rayonix	92%	12%	41%
LG36	33%	96%	30%
L498	67%	85%	82%

**Table 6:** Success rate of CNN trained with multiple datasets

	Testing		
Training dataset	Rayonix	LG36	L498
Rayonix	91%	12%	41%
Rayonix + LG36	94%	91%	54%
Rayonix + LG36 + L498	91%	94%	74%

**Table 7:** Variation of the success rate of CNN classification with respect to the size of the training data, using a combination of Rayonix and LG36 images After data augmentation is applied, the total number of images in the full set is 8000. We split this into training, validation and testing subsets in a 50/20/30% proportion, and list only the number of training images in the first column. Successive rows reduce the size of the data by random sub-selection. Our selection procedure also enforces a near-constant ratio between Hit and Miss, even as the data size is reduced. The second and third columns report the classification success when applied to the full testing sets of Rayonix and LG36 data.

Number of training data	Rayonix	LG36
4000	94%	91%
2000	92%	93%
400	93%	92%
100	91%	95%

**Table 8:** Comparison of the effect of data preprocessing techniques (simple linear contrast adjustment used in *dials.image_viewer versus* LCN) on the success rate of the CNN when applied to the Rayonix, LG36 and L498 datasets

Dataset	Simple linear contrast adjustment	LCN
Rayonix	69%	91%
LG36	95%	96%
L498	68%	81%

## Footnotes

1
https://confluence.slac.stanford.edu/display/PSDM.

2
https://www.nersc.gov.

3
https://github.com/torch/image/blob/master/doc/paramtransform.md#res-imagelcnsrc-kernel.

4 In response to a reviewer comment, we believe the best approach to increasing the classifier’s reliability for L498 (in principle) would be to increase the number of annotated images in the training set; this particular data set had the fewest number of annotated Hits (148). While preprocessing approaches may be envisioned to increase the visibility of the one-pixel Bragg spots unique to L498, such as adding a Gaussian blur to the data, this might have the unwanted effect of reducing the spatial resolution of the image. Moreover, the intent is for the CNN to discover its own optimal filter weights in a data-driven learning fashion, rather than having the experts supply the CNN with dataset-specific hints.
